# Eco-friendly production of AgNPs by ultrasound-intensified continuous method, and process evaluation via life cycle assessment and machine learning

**DOI:** 10.1016/j.ultsonch.2025.107682

**Published:** 2025-11-14

**Authors:** Juncheng Hu, Wenyu Nie, Suxu Zhao, Yong Liu, Hengyi Zhu, Shijie Tu, Jiawei Zhang, Kris Y. Yang, Ning Xue, Justin Z. Lian, Bin Dong, Stefano Cucurachi, Yuan Gao

**Affiliations:** a School of Intelligent Medicine and Biotechnology, Guilin Medical University, Guilin 541199, China; b School of Chemistry and Chemical Engineering, Nanjing University of Science and Technology, Nanjing 210094, China; c School of Engineering, China Pharmaceutical University, Nanjing 210009, China; d Institute of Environmental Science - Industrial Ecology, Leiden University, Van Steenisgebouw, Einsteinweg 2, 2333 CC Leiden, the Netherlands; e School of Traditional Chinese Pharmacy, China Pharmaceutical University, Nanjing 210009, China; f Engineering Research Center for Smart Pharmaceutical Manufacturing Technologies, Ministry of Education, China Pharmaceutical University, Nanjing 210009, China; g Department of Computer Science, University of Nottingham Ningbo China, Ningbo, Zhejiang 31500, China; h Department of Aeronautics, Imperial College London, South Kensington Campus, London SW7 2AZ, United Kingdom

**Keywords:** Ultrasound-intensified continuous method, Silver nanoparticles, Life cycle assessment, Machine learning, Eco-friendly production

## Abstract

Owing to their excellent biocompatibility and antibacterial properties, the global annual production of silver nanoparticles (AgNPs) is estimated at 400–800 tons. Therefore, developing a green and safe approach for AgNPs synthesis is urgently required. However, the conventional AgNPs manufacturing methods required toxic reductant and higher energy consumption, which makes the reaction system harmful to humans and environment. In this study, an eco-friendly ultrasound-intensified continuous system employing minimal toxic reagents was developed. The AgNPs formation process was intensified by cavitation instead of using toxic and strong reductants. Several analytical methods were conducted to systematically investigate the mechanism and performance of as-synthesized AgNPs, such as particle size distribution, zeta potential, UV–visible absorption, morphology, yield calculation, as well as *in vitro* photothermal, and antibacterial. The results clearly indicated that the physicochemical properties of AgNPs could be tuned by adjusting process parameters, including temperature, feeding rate, and ultrasonic power. Specifically, the AgNPs prepared at 90 ℃, 600 W and 50 µL/min exhibited smaller size (2–5 nm) with monodisperse, spherical shape, higher yield, enhanced photothermal, and antibacterial activities. Furthermore, life-cycle assessment (LCA) and machine learning (ML) were employed to evaluate the process sustainability and identify the key influencing parameters. Comprehensive consideration of safety, experimental results, and LCA outcomes demonstrated that the ultrasound-intensified continuous method proposed in this study is superior to the traditional NaBH_4_-based batch synthesis at the laboratory scale.

## Highlights

• Eco-friendly ultrasound-intensified continuous preparation of AgNPs was developed.
• Key synthesis parameters governed the physicochemical properties of AgNPs.
• 2–5 nm spherical AgNPs exhibited enhanced photothermal and antibacterial activity.
• Machine Learning was applied to investigate process parameter importance.
• The process sustainability was evaluated by Life Cycle Assessment.

## Graphical abstract

**Figure:**
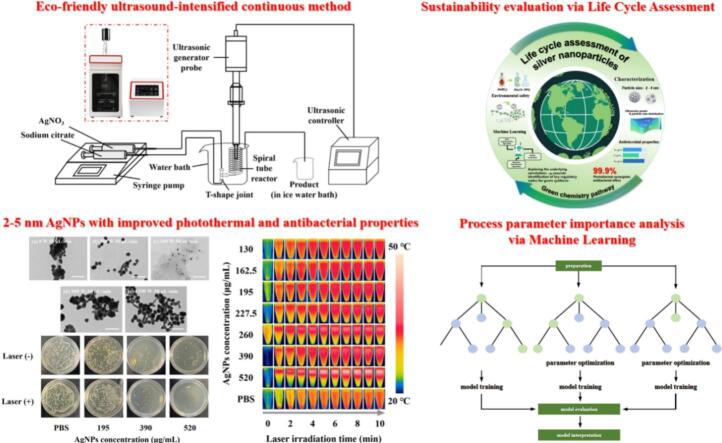

## 1 Introduction

Silver has been widely used as an antimicrobial material since ancient times due to its excellent bactericidal properties [1]. In recent decades, silver nanoparticles (AgNPs) have received increasing attention because of their enhanced antibacterial activity, non-resistance characteristics, and unique physicochemical properties, including high surface area, surface activity, and catalytic potential [[2], [3], [4]]. Consequently, AgNPs have been used as long-term antimicrobial agents in the fields of medical care, cosmetics, food storage, and textile coatings [5,6]. The global annual production of AgNPs is estimated at 400–800 tons, with its market value projected to grow from USD 2.4 billion in 2022 to USD 7.7 billion by 2030 [7,8]. The antibacterial activity and *in vivo* toxicity of AgNPs are strongly correlated with its size and morphology, with smaller particle size exhibiting stronger antibacterial activity, and spherical, mono-dispersed AgNPs providing better biocompatibility [[9], [10], [11]]. It is reported that the antimicrobial efficiency of AgNPs was highly inhibited by the particle sizes and morphology [[12], [13], [14]].

Currently, the synthesis of AgNPs is mainly achieved through physical, chemical, and biological methods [15]. These can be divided into two categories based on nanoparticle formation: top-down and bottom-up approaches [16]. The top-down method generates nanoparticles by physically breaking down bulk silver into smaller particles using techniques such as high-energy ball milling, plasma dispersion, or evaporation–condensation. Although this method avoids toxic reagents and can produce uniform particles, it is limited by its high energy consumption and low scalability [17]. In contrast, bottom-up methods rely on the reduction of silver ions into silver atoms, which subsequently nucleate and grow into nanoparticles [18,19]. To control the silver ion reduction rate and silver nuclei growth rate, as well as ensure the formation of colloidal silver, toxic reductant (such as sodium borohydride, formaldehyde, acetaldehyde, hydroquinone) are commonly used in chemical reduction method, making these processes hazardous to human health and the environment [[20], [21], [22]].

As an emerging green technology, ultrasonic cavitation has gained considerable interest for intensifying chemical reactions. During ultrasonic irradiation, cavitation bubbles form and collapse violently, releasing tremendous energy in localized high-temperature and high-pressure microenvironments [23,24]. This phenomenon enhances turbulence, micro-mixing, and mass transfer in liquids, thereby accelerating reaction kinetics and improving process stability [25,26]. In recent years, ultrasound-assisted green synthesis has emerged as an efficient and sustainable strategy for preparing AgNPs. This method utilizes plant extracts as reducing and stabilizing agents, combined with the unique physical effects of ultrasound, enabling the rapid synthesis of AgNPs with smaller sizes, narrower distribution, and enhanced bioactivity. Several studies have demonstrated the advantages of ultrasound-assisted synthesis. Gandlevskiy et al. employed Ruta graveolens extract to synthesize AgNPs with an average size of 30 nm via ultrasound assistance. Compared to traditional magnetic stirring (66 nm), ultrasound not only significantly reduced the particle size and improved monodispersity but also yielded AgNPs with significant anticancer activity. The synthesized nanoparticles were particularly effective against the B16-F10 melanoma cell line, with an IC_50_ of 2.12 µg mL^−1^ [27]. Deshmukh et al. used fenugreek seed extract to systematically compare the effects of ultrasound and magnetic stirring on the synthesis of AgNPs and iron oxide nanoparticles. Their results demonstrated that the ultrasound-assisted AgNPs were smaller, exhibited higher stability, stronger antibacterial activity, and superior antioxidant activity [28]. Nouri et al. delved into the synthesis kinetics. Using Mentha aquatica leaf extract, they found that with ultrasound, the reaction time was drastically reduced from 60 mins to 10 mins, successfully producing ultra-small AgNPs with an average size of 8 nm [29]. Moreover, the work conducted by Rajkumar and Sundar visually demonstrated ultrasound's efficacy in accelerating reactions. Using avocado seed extract for AgNPs synthesis, the solution color became dark within 15 min under ultrasound, completing the reaction within one hour. In contrast, only slightly changes were observed after one hour treatment without ultrasound. The synthesized AgNPs were successfully applied for the selective naked-eye colorimetric detection of highly toxic Hg^2+^ ions in water, highlighting their potential for environmental sensing applications [30]. Similarly, Barbhuiya et al. successfully synthesized AgNPs using turmeric extract under ultrasound, confirming their strong antibacterial efficiency [31].

As powerful data processing tools, life cycle assessment (LCA) and machine learning (ML) are applied to evaluate the process sustainability and key engineering factor in this study. ML can summarize the rules from the input variables (i.e., the variables in the experimental conditions) to the output results (i.e., the experimental results), providing predictive and interpretative insights for process optimization [32,33]. Data augmentation techniques are often applied to expand experimental datasets and enhance model robustness [34,35]. Meanwhile, LCA serves as a standardized framework to quantitatively assess the environmental impacts of nanoparticle production. For example, global AgNPs manufacturing in 2020 was estimated to generate 230–376 million kg of CO_2_ equivalent emissions [36]. Hence, greener synthesis methods are essential to reduce the carbon footprint of AgNPs production.

Based on the classical citrate-reduction method established by Lee and Meisel in 1982 (Lee-Meisel method) [5], this study introduces an ultrasound-intensified continuous-flow approach that substantially enhances nucleation and growth of AgNPs without using toxic reducing agents such as NaBH_4_. This approach enables precise control over nucleation and growth stages, enhances mixing via acoustic cavitation, reduces particle size, improves monodispersity, and eliminates the need for strong toxic reducing agents such as NaBH_4_. The use of a 5:4 M ratio of AgNO_3_ to sodium citrate follows modifications of the Lee–Meisel protocol aimed at suppressing Ostwald ripening [37,38]. Additionally, the overall sustainability and process efficiency were comprehensively evaluated using LCA and ML. This work provides a data-driven and environmentally friendly framework for the continuous, ultrasound-assisted synthesis of high-quality AgNPs.

## 2 Experimental materials and methods

### 2.1 Materials

The silver nitrate (AgNO_3_, 99 %) was obtained from Nanjing Chemical Reagent Co., Ltd. Sodium citrate (Na_3_CA, 99 %) was procured from Energy Chemical. Phosphate-buffered saline was sourced from Shanghai Sangon Biotech Co., Ltd. Pancreatic peptone and yeast extract were supplied by OXOID Ltd. Sodium chloride and sodium hydroxide were purchased from Xilong Chemical Co., Ltd. Agar powder was provided by China National Pharmaceutical Group, *Escherichia coli* ATCC 25922 was acquired from Hangzhou Baosai Biological Co., Ltd. Distilled water used in this study was provided by the GMP Training Center of China Pharmaceutical University.

### 2.2 Synthesis and charaterization of AgNPs

**Fig. 1:**
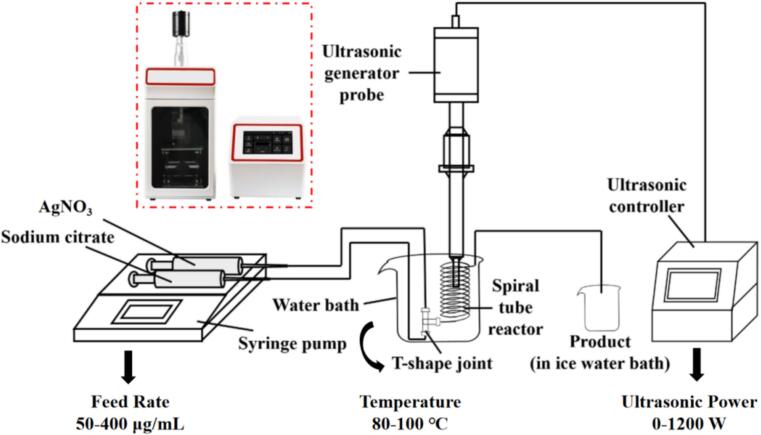
Schematic diagram of ultrasound-intensified continuous reaction system.

A custom-designed ultrasound-intensified continuous flow system was constructed following preliminary trials. The setup consists of a dual-channel syringe pump, a T-shaped mixer, and a spiral polytetrafluoroethylene (PTFE) tube reactor (inner diameter: 1.6 mm, length: 230 cm) coiled around a 28 mm diameter support. The reactor was fully immersed in a temperature-controlled water bath placed on a magnetic stirrer. An ultrasonic generator (Better-1200ST, Fangxu Technology Shanghai, China) was directly inserted into the water bath alongside the reactor to generate an ultrasonic energy field. The schematic diagram of the setup is shown in Fig. 1.

In contrast to Creighton method which employs the toxic and hazardous reducing agent sodium borohydride for AgNPs preparation [39], the present method applied green and non-polluting sodium citrate as the reducing agent based on Lee-Meisel method [38]. According to previous studies, adjusting the molar ratio of AgNO_3_ to sodium citrate to 5:4 induces digestion maturation and suppresses Ostwald ripening in the Lee–Meisel reaction. Therefore, the molar concentrations of AgNO_3_ and sodium citrate were fixed at 5 mmol/L and 4 mmol/L, respectively.

Before the experiment, the specified amounts of silver nitrate and sodium citrate were weighed and dissolved in distilled water to the required concentration. Equal volumes of these solutions were drawn into clean syringes, mounted onto the dual-channel syringe pump, and connected to T-mixer and helical tube reactor. Subsequently, the connector and reactor were submerged in the water bath, and the flow rate, bath temperature, and ultrasonic power were adjusted to the target settings, with an ultrasonic frequency of 20 kHz. During the synthesis, the reaction temperature (80 °C, 90 °C, and 100 °C), feeding rate (50, 80, 100, 150, 200, and 400 µL/min) and ultrasonic power (0, 300, 600, 900 and 1200 W) were systematically adjusted. The outlet of the reactor was immediately immersed in an ice-water bath to quench the reaction. The resulting AgNP dispersion was collected and stored at 4 °C for subsequent analysis.

The physicochemical properties of the as-synthesized AgNPs (including particle size distribution, zeta potential, UV–visible absorption, morphology, yield, and in-vitro photothermal and antibacterial performance) were comprehensively evaluated, and the detailed experimental procedures were described in Supporting Document.

### 2.3 Characterization

#### 2.3.1 Particle size distribution and Zeta potential

The AgNPs were characterized for particle size distribution and Zeta potential using a Litesizer 500 dynamic light scattering instrument from Anton Paar. Samples were equilibrated to room temperature under dark conditions after removal from a 4 °C refrigerator prior to measurement. A 1000 µL of the AgNP dispersion was transferred into a 1 cm × 1 cm plastic cuvette for automatic, aqueous-phase testing at 25 °C. In this study, the particle size distribution of the AgNPs sample was calculated using a weighted average method. For Zeta potential measurements, 200 µL of the dispersion was transferred into an electrophoretic light scattering cell ensuring contact with the measurement electrodes under the same conditions. All measurements were performed in triplicate, and results were expressed as mean ± standard deviation.

#### 2.3.2 UV–visible absorption

The UV–visible absorption peaks of the samples were characterized using a Shimadzu UV-1800 spectrophotometer. After equilibration to room temperature, 1000 µL of each sample was analyzed at 25 °C, using distilled water as the blank reference. The scanning wavelength ranged from 300 nm to 800 nm with a step size of 1 nm. The UV–vis spectra were normalized for analysis.

#### 2.3.3 Morphology

The morphology of the AgNPs was characterized using a Hitachi HT7700 transmission electron microscope (TEM). Prior to imaging, 1 mL of AgNP solution was centrifuged at 8000 rpm for 10 min, the supernatant was discarded, and the nanoparticles were resuspended in 100 µL of distilled water in a 1.5 mL centrifuge tube. The dispersion was sonicated for 10 min to ensure uniform suspension. Subsequently, 20 µL of the AgNP dispersion was drop-cast onto a copper grid and dried under an infrared lamp. The dried grid was then imaged under TEM.

#### 2.3.4 Yield determination

The yield of AgNPs under different process conditions using the mixed-enhanced microfluidic device was characterized using a Shimadzu ICPE-9000 inductively coupled plasma emission spectrometer. A 5 mL sample of AgNP solution was centrifuged at 8000 rpm for 10 min, and the precipitate was digested in 10 % nitric acid and diluted to 10 mL. The resulting solution was filtered through a 0.22 µm filter to remove insoluble impurities and stored in a 4 °C refrigerator shielded from light until analysis.

#### 2.3.5 *In vitro* photothermal and antibacterial characterization

The antibacterial activity of AgNPs against *E. coli* was evaluated in combination with their photothermal effects. Before testing, AgNP dispersions were centrifuged at 8000 rpm for 10 min, and the pellets were redispersed in PBS to obtain concentrations of 520, 390, 260, 227.5, 195, 162.5, and 130 μg mL^−1^, then shielded from light until use. The experiment could begin once the bacterial culture was grown to the logarithmic phase (OD_600_ = 0.6–0.8) and diluted 10^−^⁶ fold. A 950 µL aliquot of bacterial suspension was mixed with 50 µL of AgNP solution; PBS served as the control. with 50 μL of PBS serving as the control. The non-irradiated group was placed on a 37 °C shaker at 120 rpm, while the irradiated group was exposed to 808 nm infrared laser from 3 cm above the centrifuge tube for 10 mins. Infrared thermal images were captured at one-minute intervals and temperature changes were recorded. Following irradiation, the samples were shaken at 37 °C and 120 rpm. After 4 h of incubation, the bacterial suspension was diluted 10^-6^ fold under a sterile workbench, plated, and incubated at 37 °C for 24 h for colony counting.

### 2.4 Process evaluation via machine learning and life cycle assessment

#### 2.4.1 Process parameter importance analysis via machine learning

A hybrid model combining data augmentation and Bayesian optimization of the Random Forest Regressor (RFR) was developed to elucidate the relationship between input variables (temperature, ultrasonic intensity, flow rate, reaction time) and output (AgNP particle size). Due to the limited experimental dataset, data augmentation based on the *Nearest Neighbors* algorithm was employed to expand the dataset and improve model reliability, detailed in Table S1 [40]. The augmented data were used as the training set (80 %), and the original data as the test set (20 %). After data standardization, the Bayesian optimization algorithm was used to optimize the parameters of the RFR 50 times to obtain the best RFR model, which was then used to predict the ultrasonic preparation experimental data of AgNPs. Finally, the SHAP method and Partial Dependence Plot (PDP) method were applied to interpret the ML prediction results.

#### 2.4.2 Process sustainability evaluation by life cycle assessment

The LCA was conducted according to ISO 14040:2006 [41], encompassing goal and scope definition, system boundary, life-cycle inventory (LCI) analysis, impact assessment, and interpretation. The study focused on two lab-scale wet chemical methods for AgNPs synthesis using AgNO_3_ as the raw material, with the production of 1 mg AgNPs as the functional unit. The system boundary was defined using the gate-to-gate method, covering raw material preparation, AgNPs generation, and equipment energy consumption. LCA modeling is conducted based on the experimental steps and specific results of our study. The preparation of AgNPs was based on the specific experimental steps of this study and Mulfinger [42]. Besides, the LCA analysis was refered to Pourzahedi [43]. Based on the highest production of AgNPs in this work (90 ℃, 600 W, 50 µL/min) and previous study [44], the yields of AgNPs were defined as 71.1 % and 70 %, respectively. Due to the unequal quality of AgNPs produced by different methods, the production was normalized to 1 mg for comparison.

Open-source project Activity Browser software was applied based on Python in this study. Background data, such as chemicals and other production inputs, were collected from the Ecoinvent v.3.9 database. Since the database did not cover all chemicals, some chemicals were calculated using the chemical equilibrium method, and the missing NaH in the database was replaced with NaOH. Subsequently, the impact of the two AgNPs preparation methods on the environment was calculated and represented using EF v3.1 no LT, divided into 9 impact categories: acidification (AP), climate change (CC), freshwater ecotoxicity (FET), freshwater eutrophication (FE), marine eutrophication (ME), human carcinogenic toxicity (HCT), human non-carcinogenic toxicity (HTNC), land use (LU), and water consumption (WC).

## 3 Results and discussion

### 3.1 Effect of temperature

Temperature is one of the most critical factors influencing chemical reaction. In the traditional Lee-Meisel method for synthesizing AgNPs using sodium citrate as a reducing agent, temperature significantly impacts the resulting nanoparticles [45,46]. In this study, the influence of temperature (80, 90, and 100 °C) within the microfluidic system was investigated without ultrasound. Optical photograph, UV–vis absorption spectra, and particle size distribution of the AgNPs solution prepared under different feeding rates at different temperatures were characterized and the results were shown in Fig. S1.

As illustrated in Fig. S1a-b, AgNPs solution prepared at 80 °C exhibited minimal UV absorption (maximum absorption ∼ 0.03) and an unstable particle size distribution ranging from 5-100 nm, indicating unstable size distribution. In contrast, AgNPs sample prepared at 90 °C exhibited optical appearances consistent with existing studies [37,40], displaying strong UV absorption signals (peaking at 413 nm with a maximum of 0.99). Dynamic light scattering confirmed successful synthesis of AgNPs with size distributions ranging from 2–5 nm under various feed rate conditions. Despite literature indicating that AgNPs synthesis can occur between 60–100 °C [47,48], our result suggested that the hybrid intensified microfluidic system failed to effectively produce AgNPs using the Lee-Meisel reaction at 80 °C. This outcome might be caused by the low back-mixing characteristics of the continuous flow reaction apparatus. Since the fluid flow in the continuous flow system was predominantly plug flow, there was minimal back-mixing, and the mixing state of solutes in the solution was solely a function of reactor length [49].

This result could be explained by two main reasons. Firstly, the sodium citrate used in the Lee-Meisel method has relatively low reducing power, and typically requires boiling or near-boiling condition to proceed smoothly [38,50]. A reaction temperature of 80 °C did not provide sufficient energy for the redox reaction involving sodium citrate to overcome the activation energy barrier. Secondly, in the continuous flow reactor, mixing occurred primarily in the radial direction, with minimal axial mixing. This lead to a low local citrate ion concentration (silver ion: citrate ion ratio = 5:4), which cannot quickly reduce the silver ions, resulting in fewer nuclei at the initial stage of the reaction. Consequently, the subsequent slow reduction of silver ions tended to occur on the surface of already formed silver nuclei, causing particle growth [38]. These reasons directly manifest as weaker UV absorption peaks and larger particle sizes.

Another evidence supporting the above explanation came from the comparative experiments at 90 °C and 100 °C. As shown in Fig. S1c-d, AgNPs with a size range of 3–8 nm were successfully prepared at 100 °C. However, the corresponding UV absorption peak intensity was lower than that at 90 °C. This result can be attributed to the formation of bubbles in the reactor flow channel, which hindered the normal progression of the reaction. As illustrated in Fig. S2, the temperature reached the boiling point of the solvent (pure water) at 100 °C. Over time, tiny water vapor bubbles formed on the channel walls and gradually merged into a “bubble column”, which completely blocked the liquid flow and divided it into uneven “liquid column”. When the temperature was reduced to 90 °C, although tiny water vapor bubbles also formed over time on the channel walls, they did not reach the boiling point and subsequently dissipated back into the liquid. In summary, although increasing the temperature to 100 °C favored the Lee-Meisel reaction, the unique “bubble column” phenomenon in the continuous flow reactor hindered molecular diffusion and mixing in the solution. Although silver nuclei could form and AgNPs particles could be produced, their dispersion and concentration were lower than those achieved at 90 °C. This was reflected in the broader particle size distribution and UV absorption peak at 100 °C [51]. At 90 °C, an adequate temperature provided sufficient energy to overcome the reaction barrier without generating blocking “bubble column.” Additionally, the formation and dissipation of tiny water vapor bubbles created vortices [52], enhancing molecular mixing in the continuous flow reactor. Therefore, 90 °C was identified as the optimal temperature, providing sufficient activation energy without causing bubble-induced flow instability.

### 3.2 Influence of flow rate

The flow rate of reactants is another key factor influencing AgNP synthesis in a continuous-flow system [53]. Previous studies have shown that changes in the feeding rate in continuous flow microreactor could alter the flow patterns within the reactor and lead to the formation of vortices [27]. In this study, due to the use of a T-junction impinging flow mixer and a helical tube reactor, it was essential to investigate the impact of flow rate on the process of microfluidic controlled preparation of AgNPs.

**Fig. 2:**
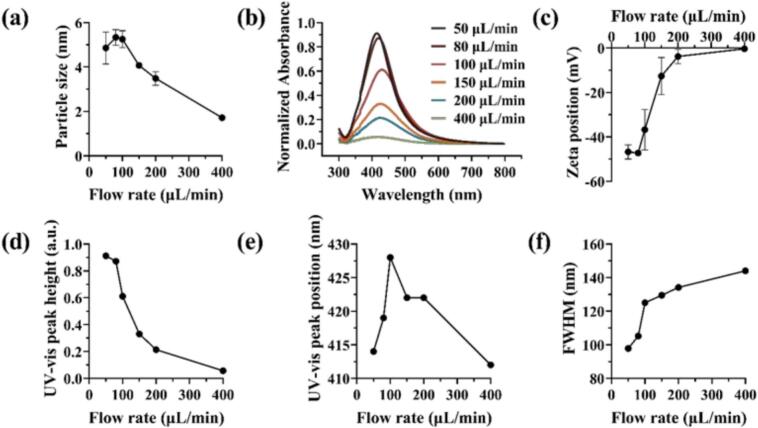
Characterization results of AgNPs prepared at 90 ℃ without ultrasound: (a) Dynamic light scattering particle size characterization, (b) UV–vis absorption peak characterization, (c) Zeta potential characterization, (d) UV–vis peak height, (e) UV–vis peak position, (f) UV–vis full width at half maximum.

Under the preparation condition of 90 °C without ultrasound, AgNPs samples were prepared by adjusting feeding rate to 50, 80, 100, 150, 200, and 400 μL/min. The effect on particle size distribution, UV–vis absorption peaks, and Zeta potential were evaluated and shown in Fig. 2.

From the results in Fig. 2, the experimental results were significantly affected by the feeding rate in the reaction device. Under the non-ultrasonic experimental condition, the AgNPs particle size increased and then decreased with the increasing of flow rate. Specifically, particle size increased from 4–5 nm at 50 µL min^−1^ to 5–6 nm at 100 µL min^−1^, then decreased to ∼ 2 nm at 400 µL min^−1^. In Fig. 2, Fig. 2, the UV–vis characterization indicated that the maximum absorption peak was around 414 nm, red-shifting to 428 nm as the feeding rate increased to 100 μL/min, and then blue-shifting to 412 nm as the feeding rate further increased to 400 μL/min. These observations were consistent with previous reports [5,38] and with the particle size results obtained from dynamic light scattering (Fig. 2a). The position of the UV–vis absorption peak, influenced by the size of AgNPs due to the unique surface plasmon resonance of AgNPs, red-shifts as the particle size increased and blue-shifts as the particle size decreased [20,38]. Additionally, the analysis of UV–vis peak height and full width at half maximum in Fig. 2, Fig. 2 demonstrated that both the UV–vis peak height and full width at half maximum of the prepared AgNPs decreased and broadened with the increasing in feed rate, suggesting the feeding rate affects the concentration and morphology of the AgNPs, as discussed in the following section.

As shown in Fig. 2c, the Zeta potential characterization indicated that the prepared AgNPs exhibited negative charge, which was related to the using of sodium citrate as the reducing agent in the Lee-Meisel method. After the reduction of silver ions, citrate ions attached to the surface of the AgNPs, forming a negative electric layer [22]. However, with increasing flow rate, the charge of the AgNPs gradually approached zero, implying potential charge instability and a tendency to agglomerate or precipitate easily when subjected to external interference. The series of experimental results above demonstrated that the feeding rate of the reaction device significantly affected the size, morphology, and zeta potential of the AgNPs, necessitating further discussion on the flow state of the reaction solution within the device.

The flow state of the fluid in the reaction device was generally characterized by the Reynolds number (*Re*) [11]. The calculation of the Reynolds number was given by the following formula:

$$\text{Re}=\frac{\rho \text{vd}}{\mu }$$

where *ρ* is the density of the fluid. In this experiment, due to the extremely low concentration of the solute, the density can be approximated as that of pure water at 90 °C, which is 965.3 kg/m^3^. The variable *v* represents the flow velocity of the fluid in the reaction device, measured in m/s, which can be calculated by dividing the volumetric flow rate by the cross-sectional area of the flow path. The variable *d* is the characteristic dimension of the reaction device, which is the diameter of the fluid flow path (0.0016 m) in this study. The variable μ is the dynamic viscosity of the fluid, approximated as the dynamic viscosity of pure water (0.0003165 Pa·s) at 90 °C.

This study Employed a helical coil reactor to induce radial vortices, the degree of secondary flow formation in the helical coil was typically quantified by the Dean number (Dn). The calculation of the Dean number was given by the following formula:

$$\text{Dn}=\sqrt{\frac{\text{r}}{\text{R}}\text{Re}}$$

where *r* is the radius of the fluid flow path, which is 0.0008 m, and *R* is the radius of the helix of the coil (0.014 m). The variable Re is the Reynolds number of the fluid.

Since the length of the pipeline in the experimental setup was fixed, variations in the feeding rate could affect the residence time of the reactants in the device, thereby indirectly influencing the reaction process. Hence, the factor of reaction time must be carefully considered. This experiment involved two reactants: silver nitrate and sodium citrate. In the helical coil reactor, the total volumetric flow rate of the reaction solution was the sum of the feed rates of those two reactants.

After calculation and measurement, the Reynolds number, Dean number, and solution retention time in the experimental reaction device were presented in Table S2. The results indicated that the entire flow system operated under laminar conditions in the absence of ultrasound. This suggested that, without additional energy input from external fields or turbulence, the driving forces of the reaction were dominated by molecular concentration gradients and inherent diffusion processes. By correlating these calculated parameters with the particle size data of AgNPs synthesized under corresponding conditions, a clear relationship was established, as illustrated in Fig. S3.

As shown in Fig. S3a, a clear correlation exists between the Reynolds (Re) and Dean (Dn) numbers and the average particle size and distribution of the AgNPs. Since the Dean number is a linear function of the Reynolds number under fixed tube diameter and helical radius conditions, both parameters exhibited similar trends in their influence on particle size. With increasing Re and Dn, the particle size initially increased and then decreased, while the particle size distribution became significantly narrower. The improved control of size distribution observed under higher Reynolds and Dean numbers was attributed to the rapid nucleation rate and uniform growth promoted by secondary vortices that enhanced radial mixing in the reaction apparatus. Conversely, at lower numerical values (Re < 10, Dn < 2), the absence of additional chemical stabilizers such as polyvinylpyrrolidone (PVP) in the reaction system limited size control, resulting in a broader size distribution. Additionally, due to the use of a T-type mixer, poor mixing efficiency at the T-joint under low numerical values (Re < 5, Dn < 1) mediated a heterogeneous nucleation process, leading to wider particle size distributions [48].

It is noteworthy that, under low numerical value, the retention time of the reactants in the reaction apparatus correspondingly increased. As shown in Fig. S3b, the actual experimental measurement results showed that the actual retention time of the device at different feeding rate was shorter than the theoretical retention time, which could be attributed to the volume expansion of the solvent under the reaction temperature of 90 °C. However, according to the actual measurement results, the trend remained consistent with the theoretical one. In the feed rate range of 50–80 μL/min, the particle size results anomalously decreased with the extension of retention time. This phenomenon could be explained by the fact that longer residence times were associated with lower flow rates, resulting in very low Reynolds and Dean numbers. Consequently, insufficient mixing within the reactor led to heterogeneous nucleation. The extended residence time also promoted Ostwald ripening, producing smaller average particle sizes but a broader size distribution. Conversely, at higher flow rates, enhanced mixing facilitated more homogeneous nucleation. However, the AgNP “seeds” generated at the T-junction were rapidly flushed out of the reactor into the ice-water bath, effectively terminating further growth. As a result, smaller and more uniform AgNPs were obtained, but the overall conversion of silver ions was reduced due to the lack of a subsequent seed growth stage. This behavior is supported by the observed decrease in UV–visible absorption peak intensity with increasing flow rate, the reduced surface modification of citrate ions in zeta potential analysis, and the lower AgNP conversion efficiency at higher feeding rates discussed later.

In summary, under experimental conditions without the application of ultrasonic energy, this experimental apparatus could successfully prepare AgNPs with particle sizes ranging from 2–6 nm. The particle size distribution and morphology of the AgNPs prepared by this method were regulated by the feed rate in the reaction apparatus. However, due to the inability to balance the need for narrow particle size distribution and high silver element conversion rate, it was necessary to apply ultrasonic energy fields to improve the process of preparing AgNPs with this reaction apparatus.

### 3.3 Influence of ultrasonic power

Extensive research has been conducted on the use of external energy fields to enhance continuous-flow reaction processes. Common methods include light radiation [54], microwave radiation [55], and ultrasonic radiation [56]. Among these method, ultrasonic radiation has been proven to positively impact the kinetics of various processes involved in crystal formation, particularly nucleation. The main impacts include: (1) Enhancing the kinetics of the nucleation process, thereby reducing the stringent conditions required for nucleation; (2) Narrowing the metastable zone of the saturation before nucleation, which facilitates lowering operational conditions and shortening the nucleation induction time; (3) Accelerating the growth process kinetics, enabling faster crystal growth and reducing the time to achieve target-sized crystals [[57], [58], [59]]. Therefore, this study employed an ultrasonic-assisted method to address the previously mentioned deficiencies in the reaction apparatus.

**Fig. 3:**
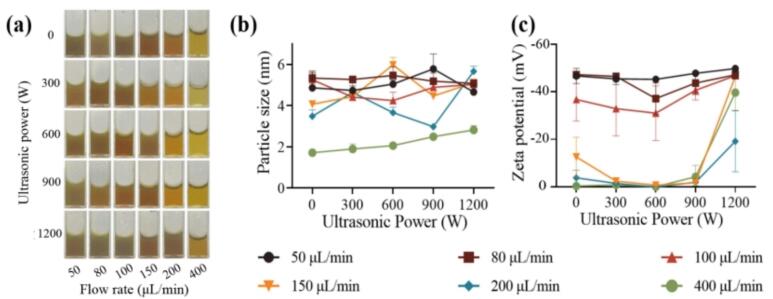
AgNPs prepared under different ultrasonic power and flow rate condition: (a) Optical photo characterization; (b) Dynamic light scattering average particle size characterization; (c) Zeta potential characterization.

Under optimal conditions at 90 °C, AgNPs were prepared by varying the flow rates and ultrasonic power, and the relevant parameters were characterized. The optical photographs, particle size, and zeta potential of the prepared AgNPs were shown in Fig. 3. Similarly, the prepared AgNPs solutions were characterized by UV–vis, and the results were shown in Fig. S4.

As shown in Fig. 3a, the solution containing spherical AgNPs exhibited the characteristic color, confirming the successful preparation of AgNPs. The color intensity was also related to the strength of the UV–vis absorption peak caused by the surface plasmon resonance of the AgNPs. Dynamic light scattering (DLS) analysis (Fig. 3b) revealed that the particle size distribution remained comparable to that obtained under non-ultrasonic conditions, ranging from 2 to 6 nm. Additionally, the variation in particle size followed a similar trend—first increasing and then decreasing with rising flow rate under the same ultrasonic power—indicating that the feeding rate was the dominant factor governing AgNP size at a fixed ultrasonic intensity. However, under different ultrasonic power condition, changes in the particle size distribution were observed, indicating that the additional energy provided by the ultrasonic waves affected the reaction process. This will be further analyzed in conjunction with the UV–vis absorption peak results. The zeta potential shown in Fig. 3c illustrated the surface charge on the AgNPs under different ultrasonic power and feeding rate condition. As the ultrasonic intensity increased, the charge amount for experimental groups with feed rates less than or equal to 100 μL/min gradually increased, while it remained relatively unchanged for groups with feeding rate greater than 100 μL/min. This result also indicated that ultrasound can influence the properties of the prepared AgNPs.

Based on the UV–visible absorption peak intensities shown in Fig. S4f, the experimental group with the highest feeding rate (400 µL min^−1^) showed a gradual increase in peak height with increasing ultrasonic power, followed by a decline at 1200 W. In contrast, the group with a moderate feeding rate (200 µL min^−1^) exhibited a gradual decrease in peak height as ultrasonic power increased, which then rose again at 1200 W. For groups with lower feeding rates, the peak intensity fluctuated irregularly with ultrasonic power.

According to the LaMer theory, a system capable of producing uniform colloids should achieve solute supersaturation within a short period at the beginning of the reaction to generate “seed” particles for subsequent crystal “growth.” This stage was known as the initial nucleation stage. After this stage, the system entered to diffusion growth stage, where reaction kinetics were driven by the concentration gradient of solute molecules in the solution, and the final particle size was determined by the remaining solute concentration in the solution [60,61].

The initial nucleation stage in this study corresponds to the mixing process at the T-shaped junction of the experimental setup, where two reactant solutions, silver nitrate and sodium citrate, collide and mix at a 180° angle, rapidly forming nanosilver “seeds”. The subsequent process of flowing into the helical tube reactor represented the diffusion growth stage. In the absence of ultrasound, the reaction kinetics were governed by the concentration gradient and eddies generated by velocity fluctuations in the flow field, as previously described. When applying ultrasonic energy, the reaction kinetics were intensified by the micromixing within high-temperature and high-pressure environment which generated by ultrasonic cavitation.

Specifically, for the previously discussed experimental groups with high feeding rate (200 and 400 μL/min), high Reynolds numbers, and low residence time under non-ultrasonic condition, rapid nucleation occured in the initial nucleation phase due to the inherent mixing intensity and eddies within the flow field. However, because of the short residence time in the helical tube and insufficient diffusion growth phase, the as-synthesized AgNPs exhibited smaller particle size, lower UV–vis absorption peaks, and lower silver element conversion rate. In contrast, when ultrasound was applied, the additional energy not only formed micro eddies at the microscopic level to promote particle growth during the subsequent diffusion growth phase, but also promoted the diffusion growth process through the high-temperature and high-pressure micro-environment created by cavitation [25,56]. This resulted in larger particle size, increased UV–vis peak height, higher particle charges, and higher silver element conversion rate. These phenomena were observed in Fig. 3b-c and Fig. S4g-h.

**Fig. 4:**
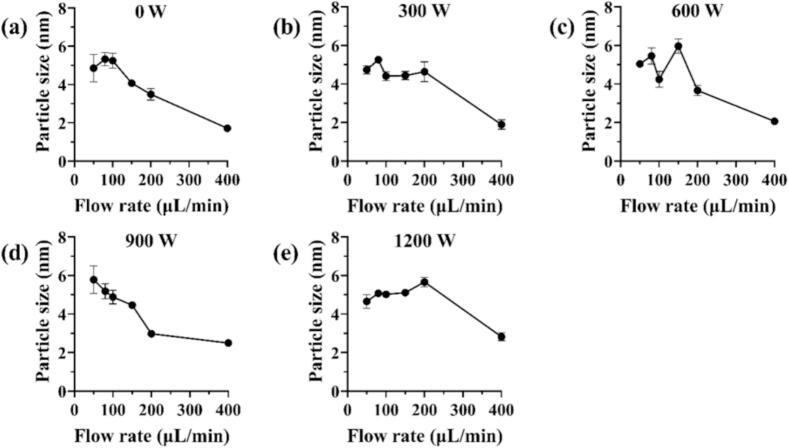
The particle size variation of AgNPs under different ultrasonic powers and feeding rate: (a) 0 W, (b) 300 W, (c) 600 W, (d) 900 W, (e) 1200 W.

For the experimental group under non-ultrasonic condition with low feeding rate, low flow regime number, and high retention time, although the T-type mixer provided intensified mixing through 180° impingement flow, the homogeneous nucleation process may not occur stably due to the reaction kinetics alone, as previously discussed. Despite using a 5:4 digestion maturation ratio of sodium citrate to silver nitrate, the Ostwald ripening process might still occur during the diffusion growth phase in the helical reactor [25]. Upon introducing ultrasonic, it was observed that as the ultrasonic power increased to 600 W, the UV–vis absorption peak height reached its maximum, while the particle size distribution (Fig. 4) and Zeta potential distribution became the narrowest. However, at either high (900 W and 1200 W) or low (300 W) ultrasonic power, the UV–vis absorption peak height and particle size distribution also varied. According to the working mechanism of ultrasound, it can propagate through the liquid in cycles of compression and rarefaction waves. At high ultrasound power, the inter-molecular attraction force could be exceeded in the medium and forming cavitation bubbles. These bubbles grow during the rarefaction phase as they absorb small amounts of vapor and subsequently collapse during compression once they reach an equilibrium size. The violent collapse of these bubbles releases substantial energy, generating both chemical and mechanical effects [62]. The lower ultrasonic power might not provide sufficient energy to disperse the potentially aggregated AgNPs nuclei in the solution, leading to a broad size distribution. In contrast, the cavitation effect induced by high-power ultrasound can generate extremely high temperature (around 5000 K) and pressure (around 500 atm), as well as high cooling rate (approximately 10^9^ K/s), along with intense shear force in the surrounding liquid medium [63,64,62]. These conditions were sufficient to cause the melting and aggregation of uniformly grown particles into larger irregular particles, or the mechanical fragmentation into irregular small particles [25,62]. The trends in UV–vis peak change (Fig. S4) and particle size distributions (Fig. 4) aligned well with the aforementioned mechanism.

**Fig. 5:**
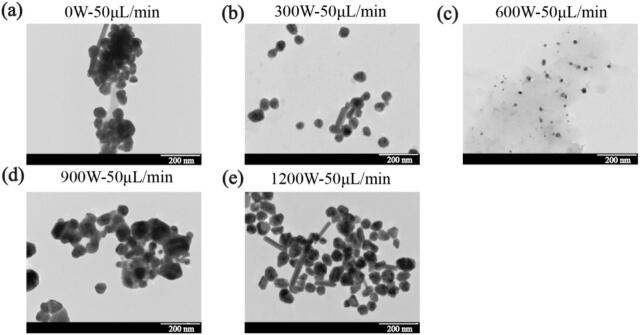
TEM images of AgNPs prepared under different ultrasonic power condition (scale bar = 200 nm): (a) 0 W; (b) 300 W; (c) 600 W; (d) 900 W; (e) 1200 W.

The TEM images in Fig. 5 were used to further verify the influence of ultrasound on AgNPs morphology. The diversity of morphologies observed under different ultrasonic conditions could be attributed to the dual role of acoustic cavitation. Under the condition of 600 W (Fig. 5c), cavitation enhanced micromixing and provided sufficient energy to promote homogeneous nucleation and spherical growth while preventing agglomeration, resulting in smaller (2–5 nm) and more uniformly distributed AgNPs [25]. This result demonstrated that proper utilization of ultrasound was able to overcome the deficiencies of the continuous flow helical tube reactor.

However, at excessively higher power (Fig. 5, Fig. 5), the extreme local condition generated by violent bubble collapse—transient temperatures up to 5000 K [26], which can induce localized melting of the nascent nanoparticles. The subsequent rapid quenching step solidified the AgNPs into irregular and non-equilibrium shapes [62]. Additionally, the powerful shock wave and shear force contributed to the fragmentation of larger particles, further broadening the morphological distribution [65]. Conversely, at lower power condition (Fig. 5b), the cavitation intensity was insufficient to dominate the process. The reaction was influenced by inherent hydrodynamic and diffusion, potentially leading to a slower nucleation rate and more pronounced Ostwald ripening effect, resulting in a slightly broader size distribution and minor aggregation tendencies compared to the optimal condition [66].

In summary, ultrasound played a crucial regulatory role in determining the morphology of AgNPs. This result further demonstrated that the proper utilization of ultrasound can effectively overcome the inherent limitations of the continuous flow helical tube reactor used in this study, enabling the preparation of smaller and more uniformly distributed AgNPs under unfavorable condition of low flow rate, small flow regime number, and long retention time, thereby achieving controllable preparation of AgNPs.

### 3.4 Yield characterization

**Fig. 6:**
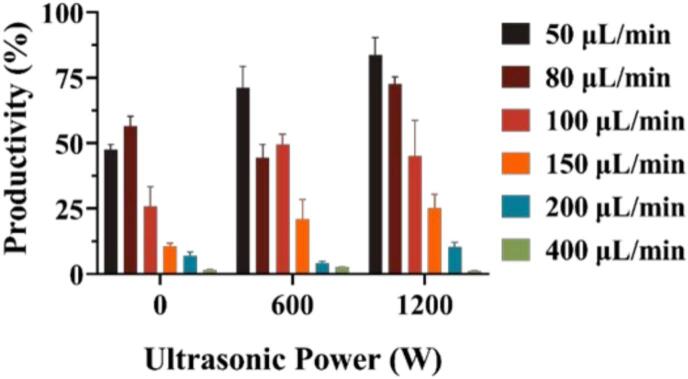
Productivity of AgNPs under different ultrasonic power and feeding rate.

Based on the previous finding, the typical ultrasound power conditions of 0 W, 600 W, and 1200 W were chosen for the experimental group. Samples were processed using the method described in Section 1.4 in Supporting Document. The silver content in the samples was calculated using the standard curve as shown in Table S3. Since two raw materials were mixed in an equal volumetric feeding rate, the theoretical yield was calculated according to the following formula to determine the final yield (Table S3 and Fig. 6).

$${\text{C}}_{\left(\text{Ideal mass concentration}\text{,}\;{\text{Ag}}^{+}\right)}=\frac{\text{0.005[MULSGN]107.8682}}{2}\text{=269.67 mg}\text{/}\text{L}$$

$$\text{Productivity}\left(\text{\%}\right)=\frac{{\text{C}}_{\left(\text{Actual mass concentration}\text{,}{\;\text{Ag}}^{+}\right)}}{{\text{C}}_{\left(\text{Ideal mass concentration}\text{,}\;{\text{Ag}}^{+}\right)}}\text{[MULSGN]100\%}$$

From the results shown in Table S3 and Fig. 6, the results clearly indicated that ultrasound significantly enhanced the yield of AgNPs. This improvement can be attributed to the effect of ultrasonic irradiation, which accelerates both nucleation and growth kinetics, thereby increasing the conversion efficiency of silver ions during synthesis. However, under the premise of requiring small size and uniform dispersion, the highest yield obtained under the 1200 W condition was not the most suitable choice. Instead, the moderate ultrasonic power of 600 W was the optimal condition for the preparation of AgNPs with monodispersed spherical morphology.

### 3.5 Photothermal and antibacterial properties

**Fig. 7:**
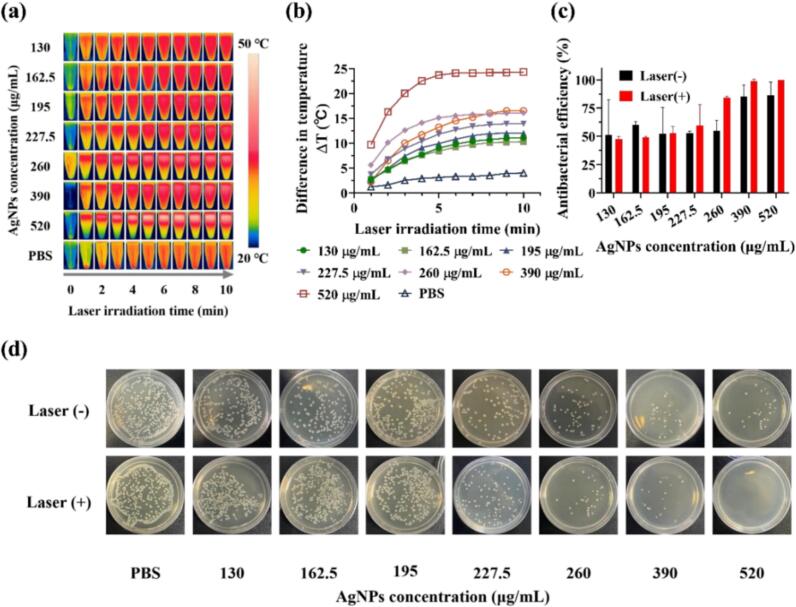
In vitro antibacterial performance of AgNPs combined with their photothermal effect. (a) Photothermal properties of AgNPs. (b) Temperature rise curve of the photothermal process. (c) The antibacterial efficiency of photothermal and non-photothermal groups. (d) Photos of bacterial solid culture medium for photothermal and non-photothermal group antibacterial experiments.

As shown in Fig. 7.a-b, the photothermal properties of AgNPs in the medium were concentration-dependent: higher AgNPs concentrations resulted in faster and greater temperature elevations. In Fig. 7. c-d, it can be observed that the minimal inhibitory concentration (MIC) of the AgNPs against *E. coli* was approximately 195–227.5 μg/mL. However, the non-photothermal minimal bactericidal concentration (MBC) was greater than 520 μg/mL, whereas the MBC concentration combined with the photothermal effect ranged between 390–520 μg/mL.

The antibacterial mechanism of the as-synthesized AgNPs involved both the intrinsic properties of silver ions (Ag^+^) and the enhanced effects conferred by near-infrared (NIR) laser irradiation. Under a dark condition, the dominant mechanism was bacteriostatic, driven by the release of Ag^+^ ions which disrupted essential microbial cellular functions. Those Ag^+^ ions can bind to thiol groups in proteins, inhibiting enzymatic activity and disrupting respiratory chains, while also causing membrane damage by generating reactive oxygen species (ROS) [3,11]. This was consistent with our experimental findings, which revealed a significant disparity between the minimum inhibitory concentration (MIC, ∼227.5 μg/mL) and the minimum bactericidal concentration (MBC, >520 μg/mL), indicating effective suppression of bacterial growth without immediate cell death.

However, upon application of NIR laser irradiation (808 nm), the antibacterial mechanism shifted to a strongly bactericidal mode due to the photothermal activation. The AgNPs exhibited excellent photothermal conversion efficiency, generating localized hyperthermia that compromised bacterial membrane integrity and induced irreversible cellular damage, including protein denaturation and DNA destruction [13,67]. This synergistic effect between releasing of Ag^+^ ions and hyperthermia resulted in a marked reduction of the MBC to 390–520 μg/mL. The enhanced bactericidal performance was attributed to the accelerated release of Ag^+^ ions facilitated by photothermally-induced membrane disruption, which promoted increased internalization of Ag^+^ ions and more severe cellular damage [68]. These findings aligned well with previous findings on photothermally enhanced AgNPs systems [69], confirming that the combination of photothermal therapy with AgNP-induced ionic toxicity offered a potent strategy for effective bacterial eradication.

### 3.6 Process parameter importance analysis via machine learning

**Fig. 8:**
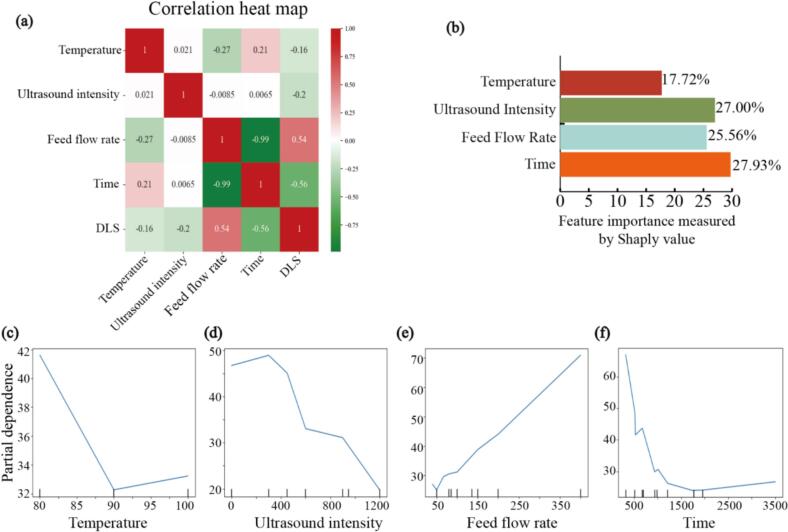
(a) The correlation between data. (b) The significance of different experimental conditions on the experimental results. (c-f) Relationship between a single experimental condition and experimental outcome.

According to Spearman, the correlation between different parameters can be observed. As shown in Fig. 8a, the correlation between Temperature and DLS was the weakest, suggesting that Temperature had the least impact on AgNPs particle size. In contrast, a strong correlation was observed between Feed Flow Rate and Reaction Time, which aligns with the actual experimental conditions. According to the interpretation of the ML model, the three factors (Ultrasound Intensity, Feeding Flow Rate, and Time) influenced the particle size of AgNPs significantly, while the influence of temperature was not significant. Therefore, further experiments on the optimal parameters between Ultrasound Intensity and Feeding Flow Rate can be designed to investigate optimal manufacturing condition. According to PDPs, Temperature, Feeding Flow Rate, and Time affected the optimal condition for the particle size of AgNPs. However, in terms of the ultrasound power, according to PDPs, the optimal condition was 1200 W, and according to the experimental data, the particle size of the 1200 W group was generally smaller than that of the 600 W group. This might be due to the limitation of the data used by ML. The 1200 W group may be damaged due to excessive Ultrasound Intensity, resulting in the best performance when measuring the particle size. Moreover, the subsequent antibacterial effect and electron microscope images were not included for comprehensive consideration. In conclusion, the ML results were relatively consistent with the experimental results, confirming the feasibility of ML in interpreting exploratory experimental data. This also demonstrated that the interpretation of ML can help researchers understand the influence of experimental parameters and formulate more detailed experimental condition.

### 3.7 Process sustainability evaluation by life cycle assessment

**Fig. 9:**
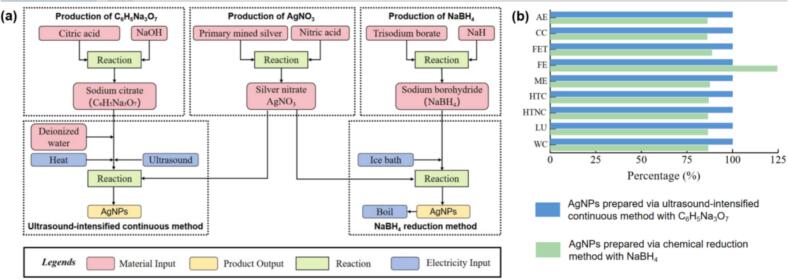
(a) System boundary flowchart of AgNPs production. (b) Relative environmental impact of AgNPs processes (with C_6_H_5_Na_3_O_7_ as 100 %).

The illustration of System boundary and the 9 environmental impact categories resulting from the two AgNPs preparation methods was shown in Fig. 9. The overall greater environmental impact was set as 100 %, and plotted it in a relative percentage format. A contribution analysis of the environmental impacts was conducted to investigate the reason. The result showed that electricity consumption was the largest influencing factor, which the electricity consumption of sodium citrate or NaBH_4_ was basically accounted for more than 95 % of the impacts. This was primarily due to the ultrasonic–heating-assisted process used in this work, which naturally requires higher electricity input than the NaBH_4_ Reduction method. In Fig. 9b, the root caused of the electricity environmental impact can be traced. It was found that the electricity was generated from hard coal, which produced many pollutants with significant environmental impact (such as CO_2_, CO, SO_2,_ etc.) [70]. Apart from electricity consumption, the next two influencing factors were the preparation of pure water and AgNO_3_, while pure water can be traced back to the fact that it also required electricity for preparation, and AgNO_3_ was due to the excessive environmental impact of silver mining that led to its higher proportion. In the FE aspect, since the pure water consumed by the NaBH_4_ path was several times that of the sodium citrate method. Since reverse osmosis was the most common method for producing purified water, organic pollutants and other contaminants may leached into the effluent during this process [71]. The subsequent treatment of pollutants may be the reason for the increase in FE.

Based on the evaluation of the LCA result and specific environmental impact quantification data, NaBH_4_ has an environmental impact of 91.06 % compared to the sodium citrate preparation. If the impact of electricity consumption was not calculated, we found that the carbon footprint of sodium citrate preparation was only 56.63 % of the NaBH_4_. Although the ultrasound-intensified continuous method used in this paper required more electrical energy, electricity was still the most easily accessible and safe resource compared to other toxic, flammable, and explosive chemicals. Besides, sodium citrate can be used as a food additive, so its safety and low toxicity were naturally more reassuring [72]. In contrast, the NaBH_4_ exhibited strong reductivity and prone to generate hydrogen during decomposition [73]. The particle size of AgNPs prepared by NaBH_4_ (2–12 nm) was larger than this study (2–3 nm). Therefore, after comprehensive consideration of safety, experimental results and LCA evaluation, it can be found that the ultrasound-intensified continuous method used in this study was superior than the AgNPs prepared with NaBH_4_ in lab scale.

## 4 Conclusion

This paper presents an eco-friendly ultrasound-intensified continuous system for the synthesis of AgNPs. The effects of feeding rate, reaction temperature, and ultrasonic intensity on the formation of AgNPs within the continuous flow system were systematically investigated to elucidate the nanoparticle formation mechanism under an ultrasonic energy field. Specifically, the AgNPs synthesized at 90 ℃, 600 W, and 50 µL/min exhibited smaller sizes (2–5 nm) with uniform and spherical morphology. These nanoparticles also demonstrated higher yield as well as superior photothermal and antibacterial properties. Subsequently, ML was employed to predict the effects of operating parameters on particle size, while LCA was applied to evaluate process sustainability. The results indicated that the chemical and electricity comsumption were the major contributors to environment impact. The key finding in this study can be summarized as follows: (1) In a continuous flow system, high temperature may not be conducive to the normal progress of aqueous reaction. Higher temperature (100°C) can cause water in the continuous flow pipeline to boil, generating bubbles that block the liquid flow, thereby interfering with the mixing of raw materials and affecting the reaction process. (2) The higher feeding rates lead to a shorter overall residence time, resulting in insufficient time for nucleation, growth, and maturation of nanoparticles after the mixture of raw materials in the T-junction. When the feeding rate is reduced to 100 μL/min or below, sufficient nucleation, growth, and maturation process can produce small-sized AgNPs. (3) Excessive ultrasound intensity can lead to the “melting” growth process of AgNPs, causing inhomogeneity during the nucleation and growth stages, which resulted in the polydispersity of the final product. (4) Combined ML and LCA analyses confirmed that the ultrasound-intensified continuous synthesis method developed in this study is superior to the traditional NaBH_4_-based batch synthesis at the laboratory scale, offering both improved performance and enhanced environmental sustainability.

## CRediT authorship contribution statement

**Juncheng Hu:** Writing – original draft. **Wenyu Nie:** Writing – original draft. **Suxu Zhao:** Writing – review & editing. **Yong Liu:** Writing – review & editing. **Hengyi Zhu:** Writing – review & editing. **Shijie Tu:** Writing – review & editing. **Jiawei Zhang:** . **Kris Y. Yang:** . **Ning Xue:** Writing – review & editing. **Justin Z. Lian:** . **Bin Dong:** Writing – original draft, Supervision, Methodology, Conceptualization. **Stefano Cucurachi:** Writing – review & editing. **Yuan Gao:** Writing – original draft, Supervision, Methodology, Conceptualization.

## Declaration of competing interest

The authors declare that they have no known competing financial interests or personal relationships that could have appeared to influence the work reported in this paper.
