# Supplementary material for: Eco-friendly production of AgNPs by ultrasound-intensified continuous method, and process evaluation via life cycle assessment and machine learning
Source: Ultrason Sonochem. 2025 Nov 14;123:107682. doi: 10.1016/j.ultsonch.2025.107682 (PMC12666576; doi:10.1016/j.ultsonch.2025.107682)
Supplement: Supplementary Data 1 [file mmc1.docx]

**Eco-friendly production of AgNPs by ultrasound-intensified continuous method, and process evaluation via Life Cycle Assessment and Machine Learning**

Juncheng Hu^a,1^, Wenyu Nie^c,1^, Suxu Zhao^c^, Yong Liu^c^, Hengyi Zhu^e^, Shijie Tu^e^, Jiawei Zhang^h^, Kris Y. Yang^h^, Ning Xue^g^, Justin Z. Lian^d*^, Bin Dong^c,f*^, Stefano Cucurachi^d^, Yuan Gao^b*^

^a^ School of Intelligent Medicine and Biotechnology, Guilin Medical University, Guilin 541199, PR China

^b^ School of Chemistry and Chemical Engineering, Nanjing University of Science and Technology, Nanjing, 210094, PR China

^c^ School of Engineering, China Pharmaceutical University, Nanjing, 210009, PR China

^d^ Institute of Environmental Science - Industrial Ecology, Leiden University, Van Steenisgebouw, Einsteinweg 2, 2333 CC Leiden, The Netherlands

^e^ School of Traditional Chinese Pharmacy, China Pharmaceutical University, Nanjing, 210009, PR China

^f^ Engineering Research Center for Smart Pharmaceutical Manufacturing Technologies, Ministry of Education, China Pharmaceutical University, Nanjing, 210009, PR China

^g^ Department of Computer Science, University of Nottingham Ningbo China, Ningbo, Zhejiang, 31500, China

^h^ Department of Aeronautics, Imperial College London, South Kensington Campus, London SW7 2AZ, United Kingdom

^*^ Corresponding author: [z.lian@cml.leidenuniv.nl](mailto:z.lian@cml.leidenuniv.nl) (Justin Z. Lian); [bin.dong@cpu.edu.cn](mailto:bin.dong@cpu.edu.cn) (Bin Dong); yuan.gao@njust.edu.cn (Yuan Gao).

^#^ Juncheng Hu and Wenyu Nie have contributed equally.

1. Characterization of AgNPs

1.1 Particle Size Distribution and Zeta Potential

The AgNPs were characterized for particle size distribution and Zeta potential using a Litesizer 500 dynamic light scattering instrument from Anton Paar. For size measurement, samples were stabilized at room temperature under light-free conditions after removal from a 4 °C refrigerator. A 1000 µL sample was transferred into a 1 cm×1 cm plastic cuvette for automatic, aqueous-phase testing at a set temperature of 25 °C. In this study, the particle size distribution of the AgNPs sample is represented using a weighted average method called Number. For Zeta potential measurements, 200 µL of the sample was transferred into an electrophoretic light scattering cell ensuring contact with the measurement electrodes under similar conditions. The particle size and Zeta potential of each sample were measured three times, with results expressed as mean and standard deviation.

1.2 UV-visible Absorption

The UV-visible absorption peaks of the samples were characterized using a Shimadzu UV-1800 spectrophotometer. The samples were equilibrated to room temperature and tested at 25 °C. Using distilled water as a reference, 1000 µL of the sample was used for measurement. The detection wavelength ranged from 300 nm to 800 nm with a step size of 1 nm. The UV-vis spectra were normalized for analysis.

1.3 Morphology

The morphology of the AgNPs was characterized using a Hitachi HT7700 transmission electron microscope. Before testing, 1 mL of the sample was centrifuged at 8000 rpm for 10 minutes, the supernatant was discarded, and the nanoparticles were resuspended in 100 µL of distilled water in a 1.5 mL centrifuge tube and sonicated for 10 mins for complete dispersion. Then, 20 µL of the resuspended AgNPs solution was dropped onto a copper grid and dried under an infrared lamp. The prepared copper grid sample was then loaded into the transmission electron microscope for image acquisition.

1.4 Yield Characterization

The yield of AgNPs under different process condition using the mixed-enhanced microfluidic device was characterized using a Shimadzu ICPE-9000 inductively coupled plasma emission spectrometer. After preparing 5 mL of the AgNPs solution, it was centrifuged at 8000 rpm for 10 min, the supernatant was discarded, and the residue was dissolved in 10% dilute nitric acid and brought to a volume of 10 mL. The resulting solution was filtered through a 0.22 µm filter to remove insoluble impurities and stored in a 4 °C refrigerator shielded from light until analysis.

1.5 *In Vitro* Photothermal and Antibacterial Characterization

This study combines the photothermal effects of AgNPs to characterize their antibacterial performance against common *E. coli*. Before testing, the AgNPs solution was centrifuged at 8000 rpm for 10 min, the supernatant was discarded, and the pellet was resuspended in PBS and adjusted to concentrations of 520, 390, 260, 227.5, 195, 162.5, and 130 μg/mL, then shielded from light until use. Once the *E. coli* culture reached the logarithmic phase and the optical density at 600 nm was between 0.6-0.8, the experiment could begin. The cultured *E. coli* suspension was diluted 10^-6^ fold, and 950 μL was aliquoted into sterile 1.5 mL centrifuge tubes, to which 50 μL of the prepared AgNPs solution was added, with 50 μL of PBS serving as the control. The non-irradiated group was placed on a 37°C shaker at 120 rpm, while the irradiated group was exposed to 808 nm infrared laser from 3 cm above the centrifuge tube for 10 mins, taking infrared thermal imaging photos every minute and recording temperature changes. After irradiation, the samples were also shaken at 37°C and 120 rpm. After 4 hours of incubation, the bacterial suspension was diluted 10^-6^ fold under a sterile workbench, plated, and incubated at 37°C for 24 hours for colony counting.

Table S1 The experimental results used for machine learning modelling

| No. | Temperature (℃) | Ultrasound intensity (W) | Feeding rate (μL/min) | Time  (s) | PSD  (nm) |
| --- | --- | --- | --- | --- | --- |
| 1 | 100 | 0 | 200 | 524.3 | 2 |
| 2 | 100 | 0 | 100 | 743 | 28.2 |
| 3 | 100 | 0 | 66.67 | 1006.7 | 2.3 |
| 4 | 100 | 0 | 50 | 1734.2 | 3.5 |
| 5 | 100 | 0 | 40 | 3499.2 | 2.4 |
| 6 | 100 | 300 | 200 | 524.3 | 3.5 |
| 7 | 100 | 300 | 100 | 743 | 36 |
| 8 | 100 | 300 | 66.67 | 1006.7 | 4.7 |
| 9 | 100 | 300 | 50 | 1734.2 | 4.3 |
| 10 | 100 | 300 | 40 | 3499.2 | 6.9 |
| 11 | 100 | 600 | 200 | 524.3 | 4.9 |
| 12 | 100 | 600 | 100 | 743 | 3.7 |
| 13 | 100 | 600 | 66.67 | 1006.7 | 4.8 |
| 14 | 100 | 600 | 50 | 1734.2 | 2.7 |
| 15 | 100 | 600 | 40 | 3499.2 | 45.2 |
| 16 | 100 | 900 | 200 | 524.3 | 35.1 |
| 17 | 100 | 900 | 100 | 743 | 82.1 |
| 18 | 100 | 900 | 66.67 | 1006.7 | 99.8 |
| 19 | 100 | 900 | 50 | 1734.2 | 2.2 |
| 20 | 100 | 900 | 40 | 3499.2 | 3.1 |
| 21 | 100 | 1200 | 200 | 524.3 | 3.4 |
| 22 | 100 | 1200 | 100 | 743 | 39.3 |
| 23 | 100 | 1200 | 66.67 | 1006.7 | 4.9 |
| 24 | 100 | 1200 | 50 | 1734.2 | 1.2 |
| 25 | 100 | 1200 | 40 | 3499.2 | 13.1 |
| 26 | 90 | 0 | 400 | 318.6 | 158 |
| 27 | 90 | 0 | 200 | 513.2 | 143.5 |
| 28 | 90 | 0 | 150 | 671.7 | 10 |
| 29 | 90 | 0 | 100 | 934.2 | 10.7 |
| 30 | 90 | 0 | 80 | 1210.7 | 11.1 |
| 31 | 90 | 0 | 50 | 1956.8 | 3.7 |
| 32 | 90 | 300 | 400 | 318.6 | 146.4 |
| 33 | 90 | 300 | 200 | 513.2 | 58 |
| 34 | 90 | 300 | 150 | 671.7 | 63.7 |
| 35 | 90 | 300 | 100 | 934.2 | 31.9 |
| 36 | 90 | 300 | 80 | 1210.7 | 8.8 |
| 37 | 90 | 300 | 50 | 1956.8 | 3.9 |
| 38 | 90 | 450 | 400 | 318.6 | 159.3 |
| 39 | 90 | 450 | 200 | 513.2 | 45.2 |
| 40 | 90 | 450 | 150 | 671.7 | 9.3 |
| 41 | 90 | 450 | 100 | 934.2 | 8.7 |
| 42 | 90 | 450 | 80 | 1210.7 | 2.2 |
| 43 | 90 | 450 | 50 | 1956.8 | 4.4 |
| 44 | 90 | 600 | 400 | 318.6 | 108.8 |
| 45 | 90 | 600 | 200 | 513.2 | 24.5 |
| 46 | 90 | 600 | 150 | 671.7 | 54.3 |
| 47 | 90 | 600 | 100 | 934.2 | 3 |
| 48 | 90 | 600 | 80 | 1210.7 | 3.4 |
| 49 | 90 | 600 | 50 | 1956.8 | 5.3 |
| 50 | 90 | 900 | 400 | 318.6 | 107 |
| 51 | 90 | 900 | 200 | 513.2 | 45.9 |
| 52 | 90 | 900 | 150 | 671.7 | 21.3 |
| 53 | 90 | 900 | 100 | 934.2 | 3.3 |
| 54 | 90 | 900 | 80 | 1210.7 | 3 |
| 55 | 90 | 900 | 50 | 1956.8 | 4.3 |
| 56 | 90 | 1200 | 400 | 318.6 | 79.7 |
| 57 | 90 | 1200 | 200 | 513.2 | 5.5 |
| 58 | 90 | 1200 | 150 | 671.7 | 1.9 |
| 59 | 90 | 1200 | 100 | 934.2 | 3.6 |
| 60 | 90 | 1200 | 80 | 1210.7 | 4.6 |
| 61 | 90 | 1200 | 50 | 1956.8 | 5.9 |
| 62 | 80 | 0 | 400 | 318.6 | 83.6 |
| 63 | 80 | 0 | 200 | 513.2 | 1.3 |
| 64 | 80 | 0 | 150 | 671.7 | 84.9 |
| 65 | 80 | 0 | 100 | 934.2 | 15.6 |
| 66 | 80 | 0 | 80 | 1210.7 | 61.9 |
| 67 | 80 | 0 | 50 | 1956.8 | 6.4 |
| 68 | 80 | 300 | 400 | 318.6 | 167.1 |
| 69 | 80 | 300 | 200 | 513.2 | 135.6 |
| 70 | 80 | 300 | 150 | 671.7 | 183.3 |
| 71 | 80 | 300 | 100 | 934.2 | 72 |
| 72 | 80 | 300 | 80 | 1210.7 | 2.4 |
| 73 | 80 | 300 | 50 | 1956.8 | 2.8 |
| 74 | 80 | 450 | 400 | 318.6 | 138.6 |
| 75 | 80 | 450 | 200 | 513.2 | 143.7 |
| 76 | 80 | 450 | 150 | 671.7 | 178.7 |
| 77 | 80 | 450 | 100 | 934.2 | 19 |
| 78 | 80 | 450 | 80 | 1210.7 | 15.8 |
| 79 | 80 | 450 | 50 | 1956.8 | 2 |
| 80 | 80 | 600 | 400 | 318.6 | 42.1 |
| 81 | 80 | 600 | 200 | 513.2 | 36 |
| 82 | 80 | 600 | 150 | 671.7 | 14.5 |
| 83 | 80 | 600 | 100 | 934.2 | 36 |
| 84 | 80 | 600 | 80 | 1210.7 | 1 |
| 85 | 80 | 600 | 50 | 1956.8 | 13.8 |
| 86 | 80 | 900 | 400 | 318.6 | 91.3 |
| 87 | 80 | 900 | 200 | 513.2 | 58.3 |
| 88 | 80 | 900 | 150 | 671.7 | 9.3 |
| 89 | 80 | 900 | 100 | 934.2 | 0.5 |
| 90 | 80 | 900 | 80 | 1210.7 | 0.8 |
| 91 | 80 | 900 | 50 | 1956.8 | 1.2 |
| 92 | 80 | 1200 | 400 | 318.6 | 2.6 |
| 93 | 80 | 1200 | 200 | 513.2 | 28.5 |
| 94 | 80 | 1200 | 150 | 671.7 | 4.7 |
| 95 | 80 | 1200 | 100 | 934.2 | 1.1 |
| 96 | 80 | 1200 | 80 | 1210.7 | 4.3 |
| 97 | 80 | 1200 | 50 | 1956.8 | 4.4 |


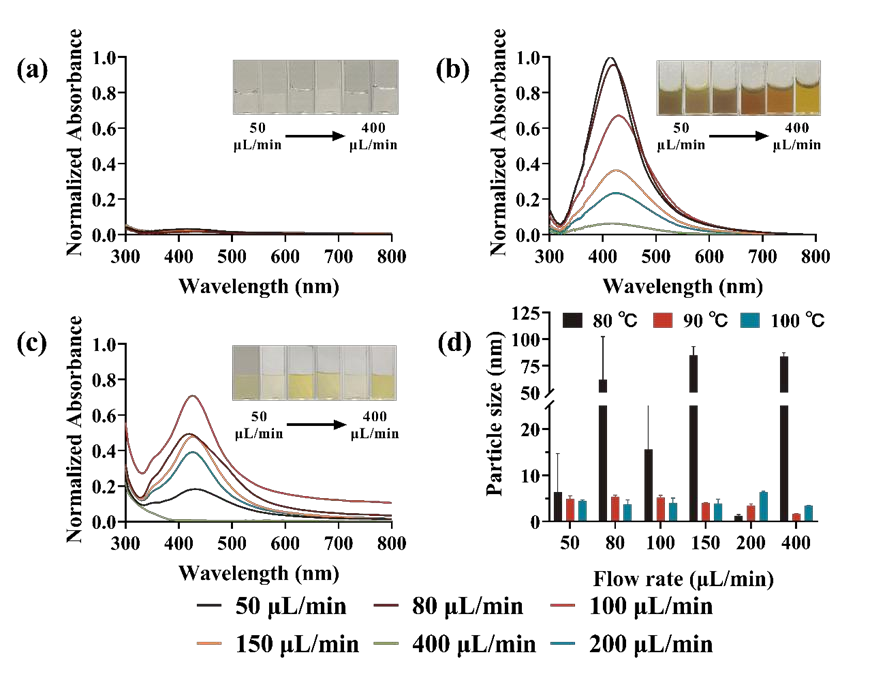


Figure S1 (a-c) Optical photograph and UV-vis absorption spectra of AgNPs solution prepared at different temperature and feeding rate without ultrasound: (a) 80 ^o^C, (b) 90 ^o^C, (c) 100 ^o^C. (d) Particle size of AgNPs obtained at 80 ℃, 90 ℃, and 100 ℃ with different feeding rate.


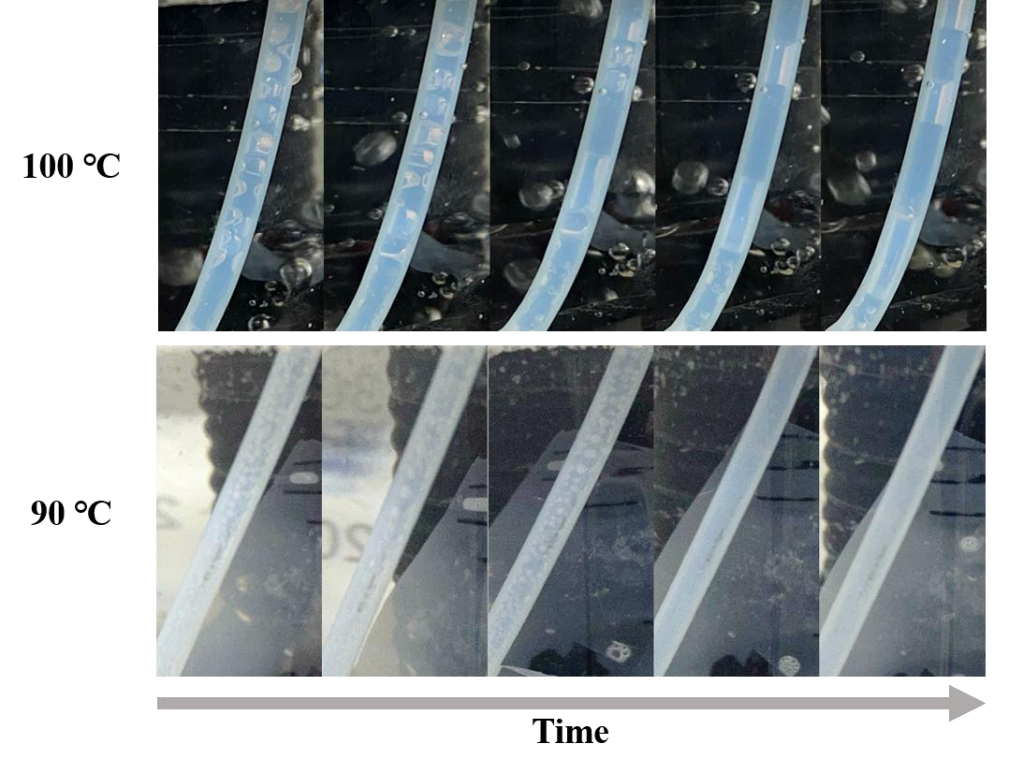


Figure S2 Bubble formation in the flow channel during the preparation process

Table S2 Dimensionless Representation Results and Solution Retention Time of Different Flow Rates at 90°C without Ultrasound

| Single channel  feeding rate (μL/min） | Total  feeding rate  (m/s) | Re | Dn | Theoretical residence time (s) | Actual residence time (s) |
| --- | --- | --- | --- | --- | --- |
| 50 | 1.67×$\text{10}^{\text{−9}}$ | 4.05 | 0.97 | 2773 | 2229±57.3 |
| 80 | 2.67×$\text{10}^{\text{−9}}$ | 6.48 | 1.55 | 1733 | 1437±115.2 |
| 100 | 3.33×$\text{10}^{\text{−9}}$ | 8.09 | 1.93 | 1387 | 1143±55.7 |
| 150 | 5×$\text{10}^{\text{−9}}$ | 12.14 | 2.90 | 924 | 759±16.7 |
| 200 | 6.67×$\text{10}^{\text{−9}}$ | 16.19 | 3.87 | 693 | 592±79.2 |
| 400 | 1.33×$\text{10}^{\text{−8}}$ | 32.38 | 7.74 | 347 | 298±15 |


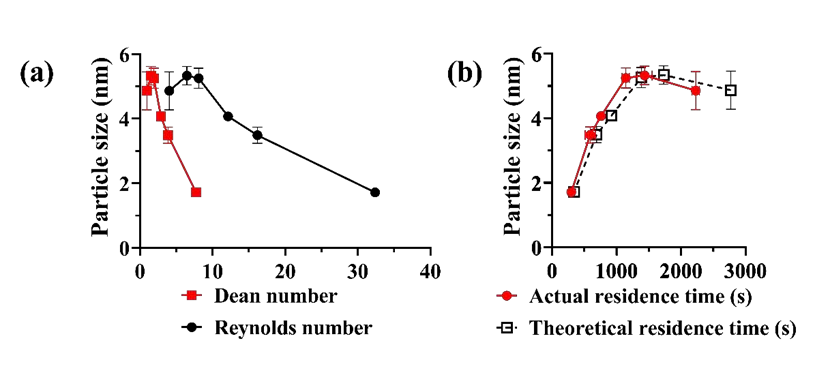


Figure S3 (a) The influence of Reynolds number and Dean number on the results of preparing silver nanoparticles. (b) The effect of different retention times on the results of nanosilver particle size.


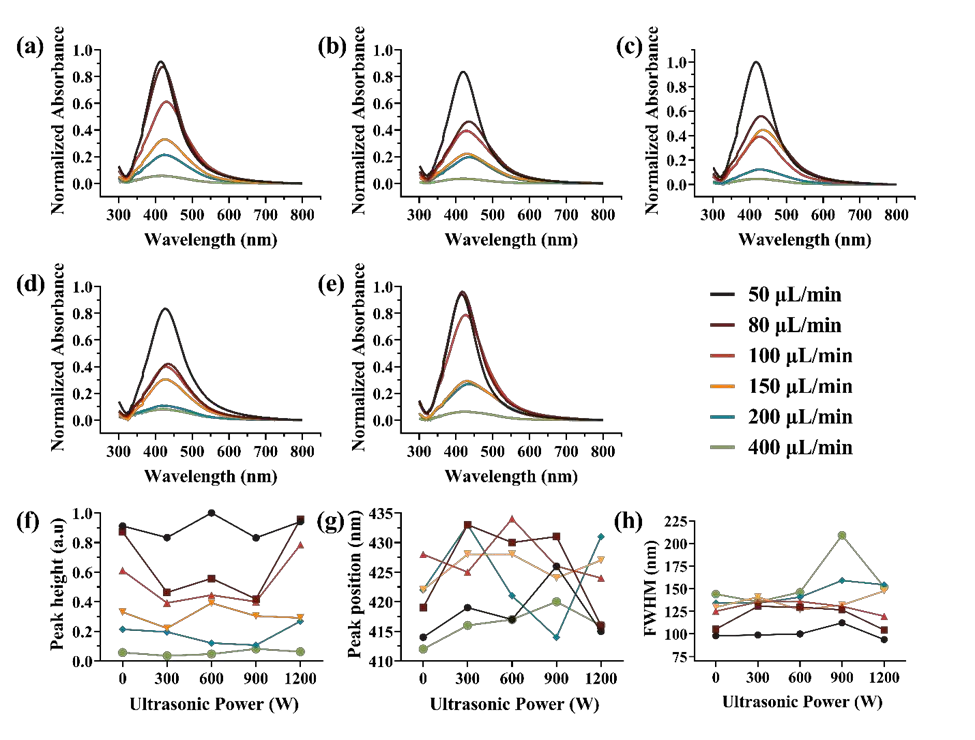


Figure S4 Characterization of AgNPs solution prepared under different ultrasonic power and feeding rate condition: (a-e) UV-vis absorption peak characterization. (f) The peak height of the UV-vis absorption peak under various condition. (g) The peak positions of UV-vis absorption peaks under various condition. (h) Full width at half maximum of UV-vis absorption peak under various condition.

Table S3 Characterization of AgNPs under different ultrasonic power and feeding rate.

| No. | Ultrasound intensity (W) | Feeding rate (μL/min) | Average Particle Size  (nm) | UV-vis | Yield (%) | Zeta potential |
| --- | --- | --- | --- | --- | --- | --- |
| 1 | 0 | 400 | 158 | 0.054 | 1.60±0.2 | -0.33±3.2 |
| 2 | 0 | 200 | 143.5 | 0.185 | 6.93±1.5 | -3.77±3.2 |
| 3 | 0 | 150 | 10 | 0.283 | 10.63±1.2 | -12.57±8.3 |
| 4 | 0 | 100 | 10.7 | 0.496 | 25.77±7.6 | -36.75±9.1 |
| 5 | 0 | 80 | 11.1 | 0.771 | 56.64±3.5 | -47.25±0.9 |
| 6 | 0 | 50 | 3.7 | 0.842 | 47.56±2.0 | -46.73±3.2 |
| 7 | 600 | 400 | 108.8 | 0.043 | 2.64±0.1 | 0.03±0.4 |
| 8 | 600 | 200 | 24.5 | 0.103 | 4.15±0.7 | 0.17±0.4 |
| 9 | 600 | 150 | 54.3 | 0.333 | 21.07±7.4 | -0.67±0.3 |
| 10 | 600 | 100 | 3 | 0.326 | 49.51±3.9 | -31.00±11.5 |
| 11 | 600 | 80 | 3.4 | 0.447 | 44.41±5.1 | -45.13±0.5 |
| 12 | 600 | 50 | 5.3 | 0.894 | 71.11±8.3 | -33.35±0.4 |
| 13 | 1200 | 400 | 79.7 | 0.057 | 1.18±0.1 | -39.67±12.9 |
| 14 | 1200 | 200 | 5.5 | 0.214 | 10.26±1.8 | -19.20±12.9 |
| 15 | 1200 | 150 | 1.9 | 0.242 | 25.22±5.2 | -46.53±1.1 |
| 16 | 1200 | 100 | 3.6 | 0.664 | 45.06±13.6 | -46.80±1.5 |
| 17 | 1200 | 80 | 4.6 | 0.864 | 72.68±2.6 | -47.17±1.7 |
| 18 | 1200 | 50 | 5.9 | 0.856 | 83.81±6.6 | -49.80±1.4 |

Table S4 R^2^ and RMSE calculated by different Machine Learning models

|  | R^2^ | RMSE |
| --- | --- | --- |
| Linear Regressor | 0.46 | 43.33 |
| KNeigbors Regressor | 0.32 | 48.47 |
| Multilayer Perceptron | 0.49 | 42.23 |
| GradientBoosting Regressor | 0.50 | 41.72 |
| Support Vector Regression | 0.47 | 42.81 |
| Decision Tree Regressor | 0.34 | 47.77 |
| Random Forest Regressor | 0.52 | 40.91 |
